# Co-creation and Evaluation of Nationwide Remote Training Service for Mental Health Education of Community Health Workers in Rwanda

**DOI:** 10.3389/fpubh.2021.632793

**Published:** 2021-08-24

**Authors:** Annik Willems, Jean-Damascène Iyamuremye, Claire Nancy Misage, Virginia Smith-Swintosky, Yvonne Kayiteshonga

**Affiliations:** ^1^Johnson and Johnson Global Public Health, A Division of Janssen Pharmaceutica NV, Beerse, Belgium; ^2^Rwanda Biomedical Centre (RBC), Ministry of Health of Rwanda, Kigali, Rwanda; ^3^Johnson & Johnson Global Public Health, New Brunswick, NJ, United States

**Keywords:** Rwanda, Community Health Workers, mental health, mobile phones, low cost, mobile health care application, technology, digital training tools

## Abstract

**Objective:** Johnson & Johnson Global Public Health and the Ministry of Health of Rwanda strengthened the mental health awareness by providing an innovative, low-cost, easily accessible, and scalable remote training service (RTS) on mental health for Community Health Workers (CHWs).

**Methods:** The RTS consisted of eight training modules shared via simple feature phones over a 4-week period. Quiz questions and baseline/endline assessments were included to assess the feasibility and acceptability of the training platform, the knowledge and self-confidence gained by the CHWs, and prospects for the sustainability of the platform.

**Results:** Ninety-three percent of the CHWs completed at least four of the eight training modules, and 42% of the CHWs improved with a higher end score. The training content was considered interesting, easy to understand, and helpful to intervene appropriately to refer patients with signs of mental illness to a hospital and to provide community and family education on mental health topics.

**Conclusion:** The RTS is feasible and acceptable for the delivery of mental health training on a large scale and contributed to strengthening the capacity in delivering mental health care at community level.

## Introduction

Rwanda, a country of 12.5 million people, is one of Africa's fastest growing economies, with an average annual growth rate of 7.2% from 2009 to 2019. In this same decade, poverty declined from 77% in 2011 to 55% in 2017, with substantial gains in major health indicators such as maternal mortality rate and life expectancy at birth (1). In 1994, the Genocide against the Tutsi took place in Rwanda, resulting in the violent death of an estimated 1,074,000 people, extensively damaged the physical infrastructure and social fabric of the country (2). Studies conducted in the years following the genocide demonstrated that genocide survivors suffer from high rates of post-traumatic stress disorder (PTSD) and other mental disorders (3).

In the immediate post-genocide period, the Government of Rwanda committed to making mental health services available to its entire population (Rwanda National Mental Health Policy) (4), and over the past 25 years has dedicated human and financial resources to the development of mental health services, with a trend toward decentralization and integration of mental health care into primary health care (5). Since 1997, healthcare facilities outside of Rwanda's capital Kigali are being equipped to treat people with mental disorders. By 2024, the Ministry of Health aims to place at least one psychiatric nurse or clinical psychologist in each of the country's 500 health centers (Rwanda Mental Strategic Plan 2020–2024). However, the need remains great at the community level as the prevalence of PTSD, depression, and anxiety is still high, with recurrent flare ups during the yearly genocide commemoration period, a 7-day period in April during which Rwandans gather to mourn the victims of the genocide (6).

Research has revealed that although mental health services are available at the national, provincial, and district levels, relatively few people seek care (data on file). In 2015, Rugema et al. highlighted numerous barriers to people seeking care for mental disorders, including fear of stigmatization, poor community awareness of mental disorders, and societal beliefs in traditional healers and prayers (7).

Community Health Workers (CHWs), the first point of contact in the community for access to health services and health education, can play an essential role in helping people access mental health services and in supporting these people in their communities. Today, ~58,454 CHWs work as volunteers for the Rwandan Ministry of Health, and national strategic plans describe planned efforts to further strengthen the capacities of CHWs to improve health care service delivery at the community level (5). The National Community Health Strategic Plan 2013–2018 acknowledges that CHWs should be appropriately trained, and that CHWs should receive training on mental illness, though in-person CHW trainings are costly and time-intensive because of the large number of CHWs to be reached (8).

Electronic health (eHealth) technology could offer a low cost, effective alternative to traditional, more resource-intensive training methodologies. There is growing interest in the use of mobile health electronic (mHealth) platforms to train and support frontline health workers in LMIC like Rwanda, with numerous examples across Sub-Saharan Africa and Asia of SMS and phone systems being successfully used to relay medical education and behavior change communications to health workers (9–12). There are fewer examples of mobile applications for the training of mental health personnel in LMIC, but there are examples of electronic training and coaching from European countries (13).

In preparation for Rwanda's 25th Genocide Commemoration in April 2019, Johnson & Johnson Global Public Health, in close collaboration with the Ministry of Health of Rwanda, engaged in a project to strengthen the mental health awareness of the country's CHW population by providing an innovative, low-cost, easily accessible, and scalable remote training service (RTS) on mental health. The training focused on supporting CHWs to recognize signs of mental illness and to provide first aid and referral advice for further professional care. This article focuses on the evaluation of the RTS platform as a contributor to community-based disease management for mental health in a LMIC (Rwanda). Specifically, we address the feasibility and acceptability of the platform, the knowledge and self-confidence gained by the CHWs, and prospects for the platform's sustainability.

## Methods

### Training Content

Content was developed from the Ministry of Health of Rwanda's existing mental health training materials. As the training was intended for CHWs, content that was salient for frontline health care workers working at community level was prioritized, with a focus on recognizing symptoms and referring people to care. Eight modules were developed, covering the following topics: introduction to common mental disorders (two parts), PTSD (two parts), depression, drug abuse, and epilepsy.

### Technical Design

The RTS was designed to remotely educate CHWs on mental health on a large scale. An Interactive Voice Response (IVR) technology delivered audio-based training modules through a simple feature phone to the CHWs across all 30 districts of Rwanda over a 4-week period. The system was powered by a mobile health platform developed by Viamo. Experience was leveraged from a similar IVR approach used to pilot training of CHWs on vaccination and Ebola outbreak response in Sierra Leone (14). The voice-based training was not designed for smart phones and did not require data connectivity.

IVR sends automated messages straight to the CHW's phone in the form of a pre-recorded voice call. All CHWs have a mobile phone that they use for SMS reporting to the Health Center using the RapidSMS application. The RTS leverages these phones to deliver an e-learning curriculum that includes a combination of mental health training voice messages and multiple-choice quizzes. Cell phones were not provided to CHWs in this project.

**Figure 1:**
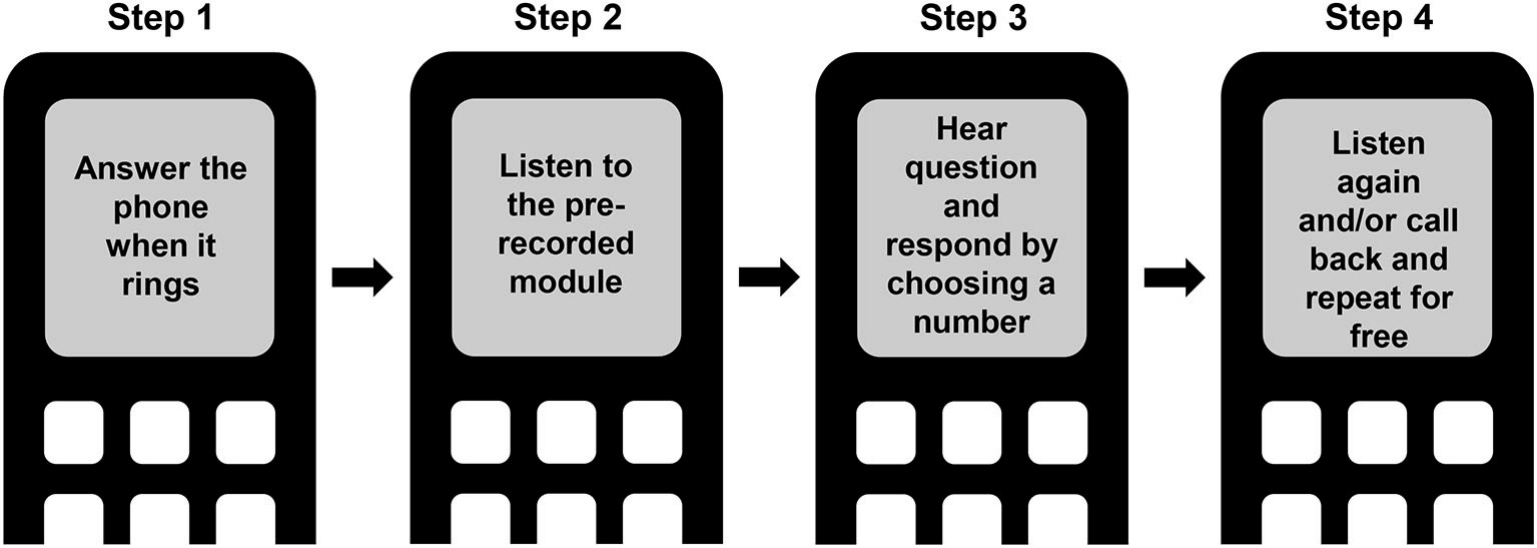
Schematic representation of the remote training service.

The CHWs received a text message alert the day before each audio-based RTS training module was to be rolled out. On the day of the training, the CHWs received a call that lasted for about 5 min. The call was composed of an introduction, a narrative, and a conclusion, followed by a quiz question. If an incorrect answer was given, the CHW heard the correct answer. After completing the quiz, CHWs were provided the opportunity to relisten to the training narrative immediately, as well as to call back (toll-free) to relisten at a later time (Figure 1). In addition, following completion of each training module, a text message was sent with the key message of the training module.

For every module, the calls were pushed to a list of CHW phone numbers provided by the Ministry of Health. The training platform included a web interface for the Ministry of Health to monitor training completion by means of a real-time dashboard.

### Pilot

A pilot with ~250 users was set up to test the RTS technical features and to refine the implementation approach. The pilot included CHWs, supervisors, and nurses in one rural and one urban area. Following the pilot, a group discussion with 12 CHWs was held to obtain feedback, including suggestions for improving the feasibility and acceptability of the RTS for the larger CHW population. Results from the pilot confirmed that the message voice was clear and that the pace allowed CHWs to retain message content. CHWs noted that their supervisors should be well-trained as they were the focal points for answering CHWs' questions. The CHWs also pointed out that the opportunity to re-listen to the narrative was very helpful as it promoted content retention.

### Procedures

In preparation for the training, the Ministry of Health organized briefings for the CHW supervisors in all five provinces to introduce the supervisors to the upcoming training program and give them guidance on how to brief the CHWs in their area of responsibility. The supervisors had an important role in encouraging the CHWs to take the training and addressing any questions.

Before the start of the main training, a baseline assessment was conducted to assess CHWs' baseline knowledge of mental health and confidence to treat patients with mental illness before they listened to any training module. The baseline assessments consisted of four knowledge questions and two confidence questions.

During the main training, the eight modules were delivered as two modules per week for 4 weeks. The spoken language of the training was Kinyarwanda (the first official language of Rwanda). The audio-based messages were intentionally kept short (5 min per module) to ensure that CHWs could listen to them amidst their busy work schedules. See Appendix 1 for the English transcripts of the training messages. At the end of each module, there was a short quiz designed for the internal team to optimize the content.

After the main training, an endline assessment was conducted to evaluate changes in knowledge and confidence after roll-out compared to baseline. The endline assessment consisted of the same four knowledge questions and two confidence questions, as well as two additional questions to assess the CHWs' satisfaction with the RTS. See Appendix 1 for the English transcript of the in-module quiz questions and Appendix 2 for the English transcript of the baseline and endline assessment questions.

The quiz and baseline/endline assessment questions were in multiple choice format; respondents could select one answer per question using a simple-feature phone keypad. Although CHWs could listen to the training message a second time, the quizzes and baseline/endline assessments could only be taken once.

A post-training discussion was organized with CHWs who had participated in the training. Discussions were held in three villages, with three to four participants in every village. Qualitative feedback was collected by asking open ended questions. There was no compensation for the participants.

### Data Analysis and Statistics

The intent-to-train (ITT) population was defined as all CHWs subscribed by the Rwandan Ministry of Health to the RTS program who listened to at least one full or partial call from the RTS system. This definition excluded phone numbers of persons who were no longer CHWs as well as numbers that were no longer in use.

The statistical analysis focused on descriptive statistics, no hypothesis testing was performed.

For the analysis of baseline and endline assessment data, the analyses were restricted to only those respondents who provided both baseline and endline data (*n* = 16,456).

## Results

### Geographic Distribution of ITT Population

Of the 55,415 eligible CHWs, 94% (51,858 CHWs) were reached by the RTS at least once (defined as the ITT population), while 6% (3,557 CHWs) were never reached by the RTS (excluded from the ITT population) (Table 1). CHWs from 30 districts, covering all five provinces in Rwanda, were represented in the ITT population.

**Table 1:** Intention To Train (ITT) population.

Population of community health workers^*^	**Number of community health workers**	**Percentage of community health workers**
Intention-to-train population	51,858	93.6
Community health workers dialed by the remote training service but never reached	3,557	6.4
TOTAL	55,415	100

* *Refers to Community Health Workers whose phone number was included in the registered list and who were automatically dialed by the Remote Training Service*.

### Training Compliance

Of the 51,858 CHWs in the ITT population, 45% (23,255 CHWs) listened to the complete training narrative of each of the eight modules, without necessarily answering the quiz questions embedded in the individual modules and/or the baseline/endline assessment questions. Ninety-three percent of the ITT population (48,204 CHWs) listened to the complete training narrative of at least four of the eight modules.

**Figure 2:**
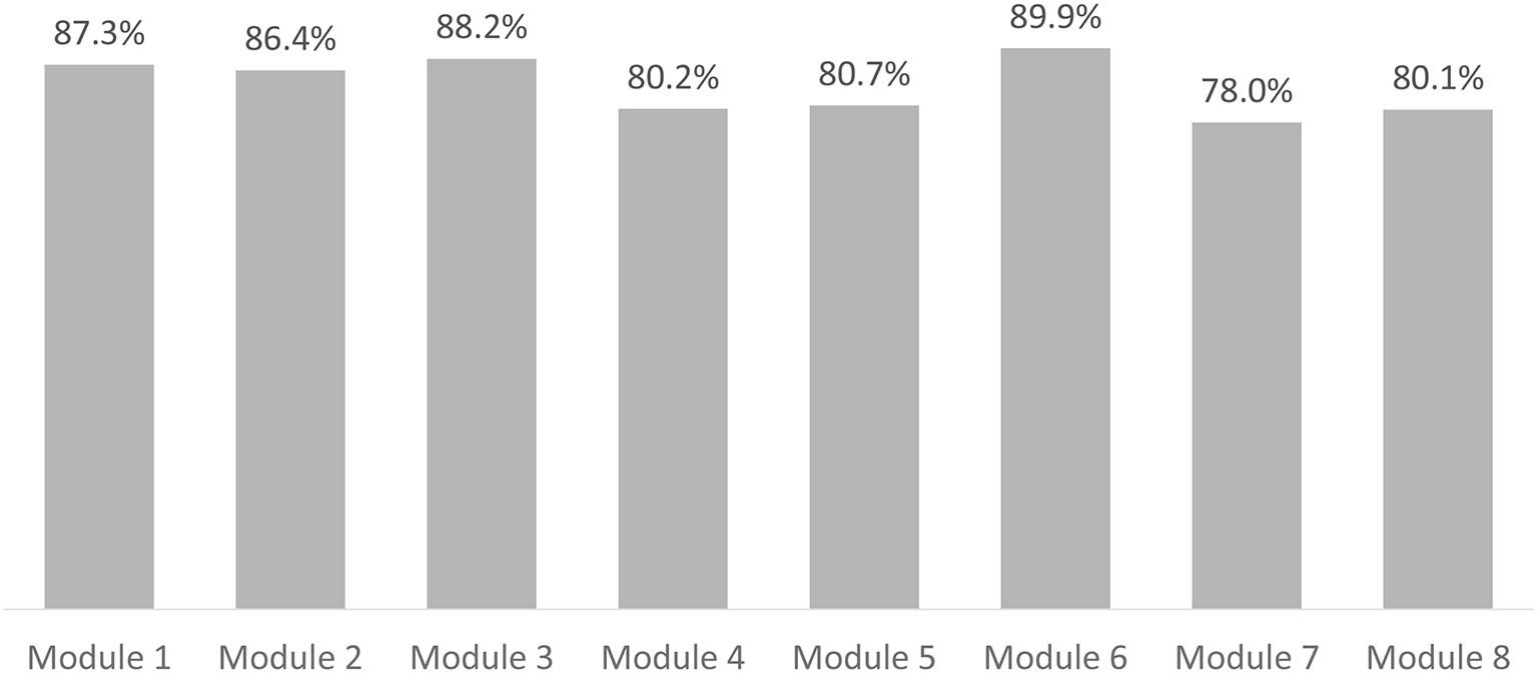
Training completion rates among intention to train population per training module.

For each of the training modules, 78–90% of the CHWs in the ITT population listened to the complete training narrative (Figure 2).

Forty-five percent of CHWs completed the baseline assessment and 59% completed the endline assessment. For the calculation for knowledge increase, we only looked at the results of CHWs who completed both baseline and endline (32%). There is a relative increase of 46% for CHWs who answered all four knowledge questions correctly at endline compared to baseline.

### Training Effectiveness: Immediate Evaluation of Mental Health Knowledge

For six of the seven in-module quiz questions, at least 91% of the respondents gave the correct answer, indicating that they understood the content of the training module. The last quiz question, concerning the contagiousness of epilepsy, was answered correctly by 81% of the respondents.

### Training Effectiveness: Change in Knowledge on Mental Health

**Figure 3:**
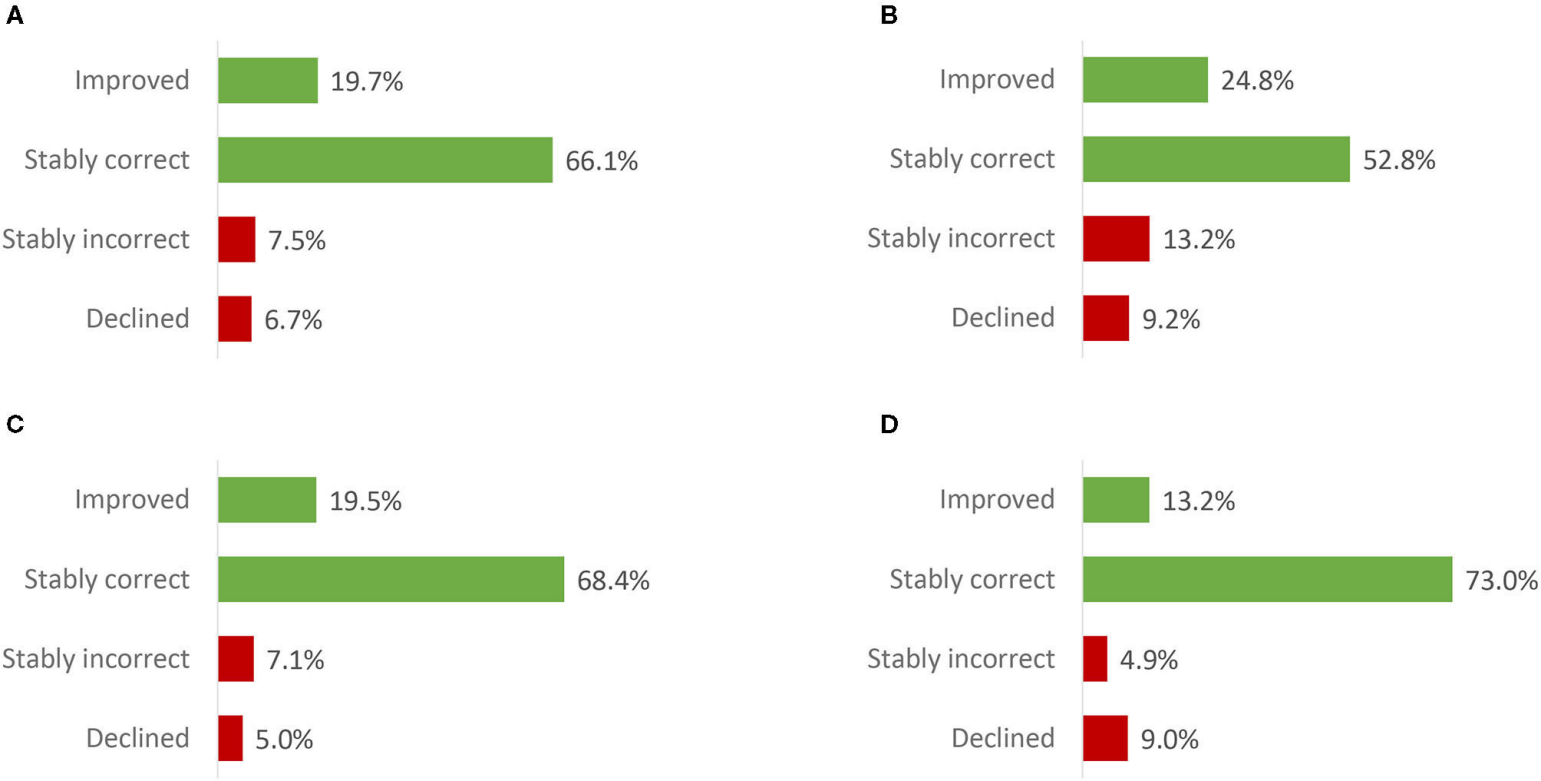
Knowledge at endline relative to baseline - within-subjects results (intention to train population). **(A)** What is a mental illness? **(B)** What is an important cause of mental illness? **(C)** How to support someone suffering from mental illness? **(D)** Is a mental illness contagious? Improved: baseline answer = “incorrect” or “unsure”; endline answer = “correct.” Stably correct: baseline and endline answers = “correct”. Stably incorrect: baseline and endline answers = “incorrect” or “unsure.” Declined: baseline answer = “correct”; endline answer = “incorrect” or “unsure.” Number of Community Health Workers that responded to the baseline and endline knowledge question [relative to the intention to train population]: 20,945 [40·4%] (Question A); 18,602 [35·9%] (Question B); 17,591 [33·9%] (Question C); 16,857 [32·5%] (Question D).

Analyses of responses to the baseline and endline knowledge questions only took into account CHWs who responded to the same question both at baseline and at endline (i.e., 33–40% of the ITT population for each question). The majority (53–73%) of CHWs included in the analysis responded correctly to the knowledge questions at both timepoints (Figure 3, “Stably correct”). The knowledge of 13% to 25% of the CHWs improved post-training, while the knowledge of 5–9% of the CHWs declined post-training. Knowledge gain was smallest for Question 4, concerning the contagiousness of a mental disease.

**Figure 4:**
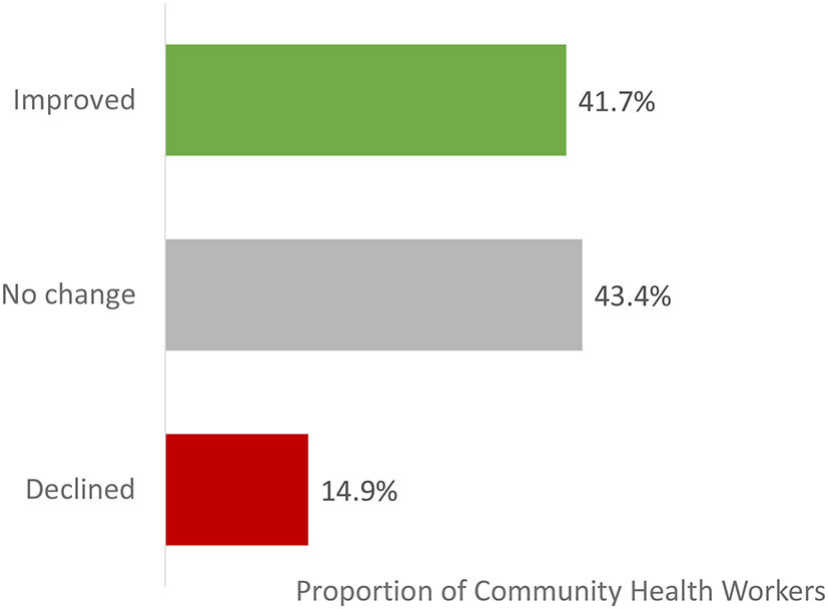
Total knowledge score at endline relative to baseline – summary of within-subjects results (intention to train population). improved: total knowledge score at endline is higher than total knowledge score at baseline. No change: total knowledge score at endline is identical to total knowledge score at baseline. Declined: total knowledge score at endline is lower than total knowledge score at baseline. The total score per participant is the total number of correct answers for the 4 knowledge questions and has a range from 0 to 4. When a participant responded “unsure” this was counted as in incorrect answer. Proportions are calculated relative to the total number of CHWs that responded to all 4 baseline and endline knowledge questions (16,856 [32·5% of the Intention To Train population]).

Analysis of the total within-subject score at baseline vs. at endline found that 42% of the CHWs had a higher total score following the course (Figure 4).

### Training Effectiveness: Change in Confidence in Providing Support to Patients Signs of Mental Illness

**Figure 5:**
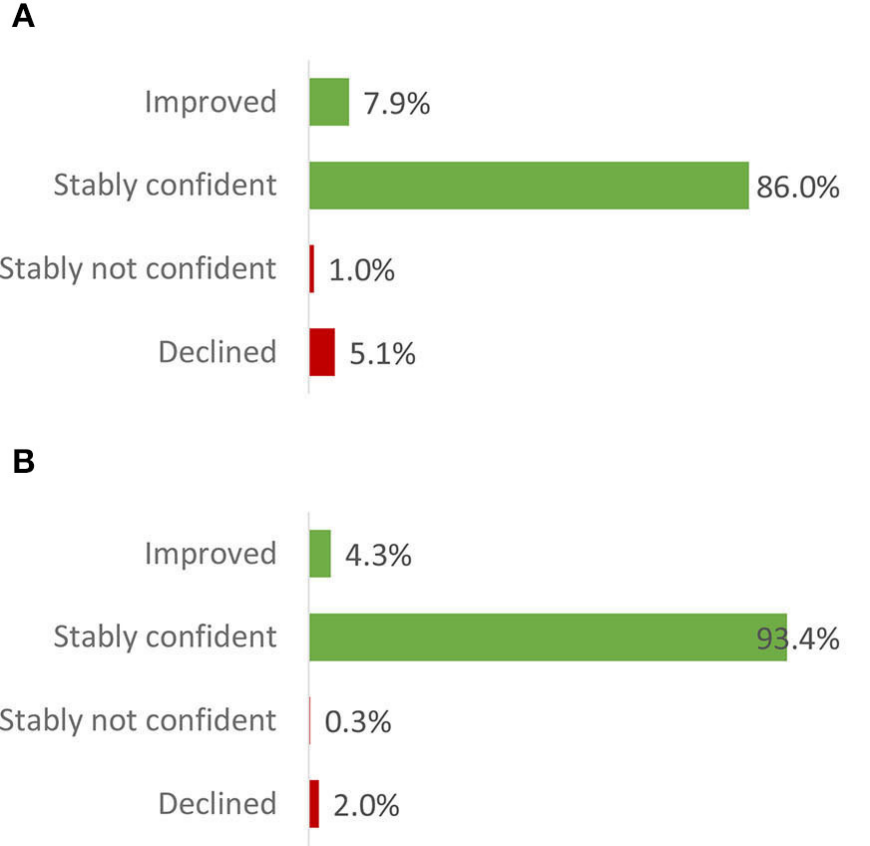
Confidence at endline relative to baseline - within-subjects results (intention to train population). **(A)** Do you feel confident to refer patients with mental illness to the hospital? **(B)** Are you confident about doing community & family education of mental health topics? Improved: baseline answer = “not confident” or “unsure”; endline answer = “confident.” Stably confident: baseline and endline answers = “confident.” Stably not confident: baseline and endline answers = “not confident” or “unsure.” Declined: baseline answer = “confident”; endline answer = “not confident” or “unsure.” Number of community health workers that responded to the baseline and endline confidence question [relative to the intention to train population]: 16,470 [31·8%] (Question A); 16,198 [31·2%] (Question B).

As for the knowledge questions, analyses of responses to the baseline and endline confidence questions only included CHWs that responded to the same question both at baseline and at endline (i.e., 32 and 31% of the ITT population for Question A and Question B, respectively). The results demonstrate that a high percentage of CHWs were confident, both before and after the training, to refer patients with a mental illness to a hospital and to provide community and family education on mental health topics (Figure 5).

### Perceptions of the Remote Training Service

In response to the two endline survey questions addressing training satisfaction, the vast majority of respondents gave very positive feedback. Specifically, 97% of respondents (29,491 CHWs) reported that they would recommend this training to others. Eighty-nine percent (27,165 CHWs) expressed interest to receive training in a similar way on other topics (data not shown).

### Lessons Learned From the Feedback Session

During a post-training discussion with 10 CHWs who participated in the training, some of the training features were praised, such as the remote nature of the course that made it very accessible to all CHWs at a time and place that fitted their schedule (one CHW noted: “The training can be done anywhere, and that was very good, very convenient”); the ability to call the service (free of charge) in case of a missed training module; the close monitoring of training participation by CHW supervisors; and the notices sent via text message to inform CHWs about upcoming trainings. The participants also expressed that the content of the course was very interesting, easy to understand, and helpful to identify PTSD during the 25th commemoration period and to intervene appropriately. The statement of one CHW highlighted the misconceptions that exist around mental health care and underscored the value of this training, “The knowledge on symptoms helped to differentiate Mental Illness from demon possession and to explain to the families to go to a health center and not to church or traditional healers.”

During the group discussion, examples were given about the impact of the training in relation to CHWs' daily work. CHWs reported that before the training they did not know that patients with mental health issues could be treated at the hospital, and this seems at odds with their reported high level of confidence at baseline that they could refer patients with mental disorders to the hospital.

Several recommendations were proposed for further implementation and improvement of the RTS. The CHWs were interested in receiving training in a similar format on other topics, in particular drug abuse. The CHWs considered it difficult to answer the questions using their mobile phone keys. Although the duration of the modules (~5 min) was considered acceptable, they reported that 10 min per module would be more appropriate as it would permit provision of more in-depth information. They also recommended opportunities to ask questions about the training. To facilitate application of new knowledge on mental health, they also strongly recommended that village leaders receive the same training, so they would support the CHWs in their decisions to refer patients with mental illness symptoms to a hospital.

## Discussion

The relatively high prevalence of mental health disorders in Rwanda, largely due to the 1994 Genocide against the Tutsi, has triggered the Rwandan Ministry of Health to invest in mental health care and to increase citizens' awareness about mental health (5). Given the important role of CHWs in extending primary health services to communities, it is essential to provide mental health education to the large group of ~58,454 CHWs across Rwanda to help increase use of mental health services and to decrease the stigma of mental illnesses.

An RTS was set up to refresh or expand the CHWs' basic knowledge of mental health disorders and strengthen their capacity to recognize symptoms of mental health problems. The endline assessment included questions to evaluate whether the selected IVR technology was well-accepted by CHWs. In addition, knowledge and confidence questions were posed at baseline and endline to assess whether the training succeeded in increasing mental health awareness and confidence to educate community members and refer those with symptoms to other services.

Based on the data gathered from the endline satisfaction questions and the post-training feedback session, we conclude that CHWs found the RTS to be highly feasible and acceptable as a training platform. In further support of this conclusion, the completion rates per module remained stable across the modules, with at least 78% of the ITT population listening to the complete training message per module. This indicates that the CHWs maintained their interest and engagement over the 4-week period. Our findings are consistent with other research that has shown that mobile technology interventions to improve the range and quality of services provided by CHWs can be feasible and acceptable (15). They are also consistent with research showing that a standardized mental health training for CHWs in South Africa can improve knowledge, confidence and attitudes (16).

The present study's findings on knowledge from the baseline and endline assessments indicate that the training platform enabled CHWs to refresh or acquire knowledge about mental health. Forty-two percent of the CHWs that responded to all of the baseline and endline knowledge questions demonstrated knowledge gain over the course of the training. Assuming that each CHW, on average, reaches ~23 patients per year with a mental illness in his/her community (data on file), these 7,032 CHWs who exhibited overall knowledge improvement would be able to provide better community-based support for 161,736 patients with a mental health disorder. The decline in knowledge in a small number of CHWs is interesting and warrants further research. The results of the quiz and the baseline and endline responses also reveal that misconceptions remain concerning contagiousness of mental illnesses, particularly epilepsy. This may indicate that the training module covering this topic was more difficult to understand, that there is a strong (erroneous) belief about contagiousness that is difficult to change and/or that the assessment questions were unclear. Important advantages of the RTS were identified, in particular the flexibility it offers CHWs as to where they take the training, eliminating the need for travel, and as to when they take the training, allowing accommodations to personal schedules. These factors help reduce logistical and cost barriers to training, leading to better training reach and in turn contributing to a more educated CHW population.

The group discussions with the CHWs also revealed that listening to some of the modules led CHWs to initiate discussions with other CHWs or their supervisors about mental health. This suggests that the RTS can have a ripple effect, even when CHWs are not exposed to all modules.

RTS is an economical intervention. The total cost of the RTS intervention, including development, piloting, and training of the CHWs is ~2.50 USD per CHW, excluding the cost of obtaining a cell phone.

However, the use of mobile technology to deliver training in short voice files does not obviate the need for classroom training (17). Recent research has found that the implementation of the mHealth solution RapidSMS, a two-way communication system between CHWs and the Ministry of Health in Rwanda, increased the use of maternal and child health services, but only when combined with additional support and as part of a comprehensive intervention (18). The RTS platform should therefore be considered as a means to further build on the knowledge gained in face-to-face, classroom trainings.

The present study had several limitations. For the delivery of the training service, the Ministry of Health provided a list of registered CHWs with their mobile phone numbers. About 6% of the registered CHWs were never reached by the IVR technology, suggesting that the list of phone numbers may not have been up-to-date. To facilitate optimal training implementation, an updated list of CHW mobile phone numbers should be created and maintained. The baseline and endline assessment were not mandatory and this resulted in many missing data points. Another limitation of the baseline and endline assessment design was that only CHWs with both scores were analyzed, which raises bias issues. Our intent was to make individual CHW comparisons between pre- and post- test overall scores and was why we only included matched pairs.

Several factors could have resulted in missing outcome data, including failure to complete training modules and technical challenges with the quizzes and baseline/endline assessments. Based on the CHW feedback session, it appears likely that many of the missing responses were due to technical issues; for example many CHWs with a feature phone did not know that when listening to a call, they must activate the keyboard by pressing a key *before* pressing a key to answer a question. Missing outcome data could also have resulted from the way the questions were posed, as others have described the need for careful design of questions to ensure clarity and to minimize cognitive burden for respondents, many of whom may not have prior experience in taking automated surveys (19). The large amount of missing outcome data might indicate that the RTS is a good platform to share information but is not as well-suited for collecting data, at least among certain populations.

There also were a number of confounding factors, notably that any previous mental health trainings in which CHWs had participated, the length of time they had been working as a CHWs, if they were linked to a health center with a psychiatric nurse or other mental health staff, or their level of education were not noted, but could represent bias. In the group discussions, CHWs stated they did not know that mental health patients could be referred to a hospital. At baseline, however, CHWs report high scores related to confidence to refer patients with signs of mental illness to a hospital. This might be due to social desirability bias or response-shift bias.

The RTS platform was designed for large scale use and allows the Ministry of Health to continue using the system beyond commemoration for other training topics. Currently, the study partners are exploring pathways to make the RTS platform an integral part of Rwanda's capacity-building for CHWs. Specifically, the authors are investigating options for storing the current training content in a way that will allow any interested party to access it. The Ministry of Health is also considering how the RTS platform might be used for other training topics. Negotiations are underway to use the platform for training on Ebola psycho-social support. RTS could be used to provide awareness or refresher training to diverse populations, on diverse topics, and could potentially be rolled out in other developing countries that could benefit from remote training services.

In conclusion, the available data demonstrate that Rwandan CHWs found the RTS to be a feasible and acceptable tool to supplement existing CHW training approaches, and that the tool strengthened mental health awareness and self-confidence among the CHWs. Further research is needed to evaluate the impact of RTS on patient outcomes. As a result of mental health awareness campaigns, including the RTS, health centers in Rwanda have requested that CHWs report monthly on their number of referrals for mental health care. The new indicator is in the process of being integrated. The Ministry of Health will also be measuring a change in accessing care from the time of the RTS training based on data from their Health Information Management System.

## Data Availability Statement

The original contributions generated for the study are included in the article/Supplementary Material, and further inquiries can be directed to the corresponding author/s.

## Ethics Statement

Ethical review and approval was not required for the study on human participants in accordance with the local legislation and institutional requirements. The ethics committee waived the requirement of written informed consent for participation.

## Author Contributions

AW drafted the Article with support from Leen Mathys, a medical writer affiliated with Janssen Research and Development. Anne Depaepe, an employee of Johnson & Johnson, ran the statistical analyses. Sonia Pelletreau an employee of Johnson & Johnson and provided editorial input. All authors contributed to the conception and design of the RTS. The article was revised with substantial input from all authors, who approved the final manuscript.

## Conflict of Interest

The authors disclose receipt of the following financial support for the research, authorship and publication of this article: funding from Janssen Pharmaceutica NV and the Rwandan Ministry of Health.

## Publisher's Note

All claims expressed in this article are solely those of the authors and do not necessarily represent those of their affiliated organizations, or those of the publisher, the editors and the reviewers. Any product that may be evaluated in this article, or claim that may be made by its manufacturer, is not guaranteed or endorsed by the publisher.
