# Supplementary material for: Co-creation and Evaluation of Nationwide Remote Training Service for Mental Health Education of Community Health Workers in Rwanda
Source: Front Public Health. 2021 Aug 24;9:632793. doi: 10.3389/fpubh.2021.632793 (PMC8423103; doi:10.3389/fpubh.2021.632793)
Supplement: Supplementary file 1 [file Data_Sheet_1.PDF]

Supplementary Material

Appendix 1: Content of the Training Messages and In-module Quiz Questions

Module 1 - Introduction to Common Mental Disorders

**Segment**	**English translation of training message**
Introduction	Greetings! This is an important message from the Ministry of Health regarding training about mental health. This training will focus on understanding common mental illnesses and how to support affected people.<br><br>The training will last for four weeks during which you will be receiving calls twice a week. Each call lasts for less than five minutes.<br><br>Please, listen carefully to the entire call and answer the quiz by pressing digits on your phone.
Lesson Outcome	Today we will discuss about mental health and the common mental disorders.
Narrative	Mental health includes the emotional, psychological, and social well-being. It affects how people think, feel, and act. It also helps determine how people handle stress, relate to others, and make choices<br><br>The most common mental disorders are trauma, depression, epilepsy, drug abuse and others.<br><br>If you wish to listen to this message again, press 1.
Conclusion	This is the end of today's lesson. After this lesson you will receive an SMS that summarizes what you have learned in today's session. If you wish to re-listen to this message or to share it with someone, you can call this number [Phone number]. It is free of charge.

	Thank you for listening to this message. Next time we will ask you a few questions related to your general knowledge on mental health (Baseline questions).

Module 2 - Mental Disorders I

**Segment**	**English translation of training message and in-module quiz question**
Advance SMS	Greetings. The Ministry of Health will call you tomorrow in the afternoon for the training about common symptoms of mental health disease. You will be called by [Phone number]. Thank you.
Introduction	Greetings! This is an important message from the Ministry of Health regarding training about mental health.<br><br>Listen carefully to the entire call and answer to the quiz.
Lesson Outcome	Did you know some of the symptoms of mental disorders?
Narrative	Some of the symptoms of mental health disorders are change in behavior, extreme mood changes, excessive fears or worries, etc.<br><br>E.g.,<br>● Isolation, lack of sleep, feeling of excessive fear<br>● Reliving past traumatic moments<br>● Agitation, hyperactivity, irritability, fighting<br>● Self-talking or laughing with no reason<br>● Hallucinations<br>● Attempting or committing suicide etc.<br>Mental disorders may be caused by various factors such as:<br><br>● Every day’s life challenges / problems (examples: abuse, extreme poverty, war, exile, etc)

	- Changes in one's life (loss of a loved one, loss of valued properties, disasters like earthquake, flood, genocide, etc) - Family history of mental illnesses (heredity) - Brain hormones - Drug abuse, etc If you wish to listen to this message again, press 1.
Closing	Please listen to the quiz.
Quiz/Test	Please answer one question related to today’s lesson:<br><br>A person with a mental illness presents signs of changes: - In comportment - In oral expression - In thinking - In interaction with others If yes, press 1. If no, press 2
Correct Answer	Thank you, you've responded well.
Incorrect Answer	Thank you for trying, the correct answer is “Yes”
No response	You have not responded to this question. Please listen to the question again and press a number that corresponds to your answer choice.
Conclusion	This is the end of today’s lesson. After this lesson you will receive an SMS that summarizes what you have learned in today’s session. If you wish to re-listen to this message or to share it with someone, you can beep this number [Phone number]. It is free of charge.<br><br>Next time we will talk about the basic first aid that can be provided to a person that shows signs of mental disorders, how to refer him and how educate the community and the family to take care of someone with mental disorders.

Post call SMS (Key Messages)	Some of the important symptoms of a mentally-ill person are the changes of comportment, of oral expression, of thinking and of social interaction with other individuals.<br><br>Mental illnesses may be caused by various factors such as: social problems of every day's life (examples: abuse, extreme poverty, war, exile, etc).

<u>Module 3 - Mental Disorders II</u>

**Segment**	**English translation of training message and in-module quiz question**
Advance SMS	Greetings. The Ministry of Health will call you tomorrow in the afternoon for the training about on how to provide first aid for mental health patient. You will be called by [Phone number]. Thank you.
Introduction	Greetings! This is the important message from the Ministry of Health regarding training about mental health.<br><br>Listen carefully to the entire call and answer to the quiz.
Lesson Outcome	Did you know how to offer the basic first aid to a person that has symptoms of mental disorders? Did you know how you can educate his/her family?
Narrative	It is important to listen to a patient suffering from mental disorders. You do that by reassuring them, and when talking to them, don't be judgmental.<br><br>It is key to advocate for the patient's access to basic healthcare needs such as medical insurance. It's also your responsibility to contribute to discrimination and stigma reduction within the community.<br><br>If you wish to listen to this message again, press 1.
Closing	Please listen to the quiz.

Quiz/Test	Please answer one question related to today's lesson:<br><br>A person that shows signs of mental illness should be taken<br><br>● To a church<br>● To a witch doctor<br>● At the hospital<br><br>If it's to a church, press 1. If it's to a witch doctor, press 2. If it's at the hospital, press 3.
Correct Answer	Thank you, you've responded well.
Incorrect Answer	Thank you for trying, the correct answer is "At the hospital".
No Response	You have not responded to this question. Please listen to the question again and press a number that corresponds to your answer choice.
Conclusion	This is the end of today's lesson. After this lesson you will receive an SMS that summarizes what you have learned in today's session. If you wish to re-listen to this message or to share it with someone, you can beep this number [Phone number]. It is free of charge.<br><br>Thank you for listening to this message. Next time we will talk about causes and symptoms of PTSD.
Post call SMS (Key Messages)	It is primordial to listen to a mentally-ill person, to reassure and talking patiently to him/her and not judging him/her.<br><br>Helping the family members of the mentally-ill person to transport him/her at the hospital is important.<br><br>It is necessary to continue caring for him/her and to be his/her advocate at every level.

Module 4 - PTSD I

**Segment**	**English translation of training message and in-module quiz question**
Advance SMS	Greetings. The Ministry of Health will call you tomorrow in the afternoon for the training about PTSD and the symptoms. You will be called by [Phone number]. Thank you.
Introduction	Greetings! This is the important message from the Ministry of Health regarding training about mental health.<br><br>Listen carefully to the entire call and answer to the quiz.
Lesson Outcome	Did you know the causes and the most important signs that can help you recognize a person suffering from trauma, as well as how to help him/her?
Narrative	Some of the causes of PTSD are being abused or witnessing horrors. Examples: genocide, wars, abuse, violence, natural disasters, etc.<br><br>However, this doesn't mean that every person that has encountered these events has PTSD.<br><br>Some of the most important signs that shows a person with PTSD are: reliving horrible moments from the past, having nightmares, avoiding anything that could remind them of a horrible event from the past, startling with fear at any occasion and lacking sleep.<br><br>You can support someone suffering from PTSD by listening to them, comforting them, and by avoiding to judge them.<br><br>You should also educate the family members to take care of the patient and to take him/her at the hospital.<br><br>If you wish to listen to this message again, press 1.
Closing	Please listen to the quiz.
Quiz/Test	Please answer one question related to today’s lesson:<br><br>PTSD can be caused by being abused or witnessing horrors such as a genocide, war or abuse.

	If Yes, press 1. If No, press 2.
Correct Answer	Thank you, you've responded well.
Incorrect Answer	Thank you for trying, the correct answer is “Yes”.
No response	You have not responded to this question. Please listen to the question again and press a number that corresponds to your answer choice.
Conclusion	This is the end of today’s lesson. After this lesson you will receive an SMS that summarizes what you have learned in today’s session. If you wish to re-listen to this message or to share it with someone, you can beep this number [Phone number]. It is free of charge.<br><br>Thank you for listening to this message. Next time we will talk about the first aid that you can provide to a person with PTSD.
Post call SMS (Key Messages)	Some of the most important signs that shows a person with trauma are reliving horrible moments from the past, having nightmares, avoiding anything that could remind of a horrible event from the past, startling with fear at any occasion and lacking sleep.

<u>Module 5 - PTSD II</u>

**Segment**	**English translation of training message and in-module quiz question**
Advance SMS	Greetings. The Ministry of Health will call you tomorrow in the afternoon for the training about mental health. You will be called by [Phone number]. Thank you.
Introduction	Greetings! This is the important message from the Ministry of Health regarding training about mental health.<br><br>Listen carefully to the entire call and answer to the quiz.

Lesson Outcome	Did you know where to refer a person with PTSD, did you know how to support, and to provide follow-up?
Narrative	After you've already provided first aid to a person experiencing PTSD, you should encourage him/her to seek out help from the healthcare facilities. You should encourage his/her family to support him/her to get to the hospital.<br><br>If the condition worsens, you should immediately help him/her to get to the nearest health center.<br><br>For a person under medical treatment, you can visit him/her, encourage him/her to follow the doctor's instructions and to adhere to the treatment.<br><br>It is important to visit a family that has a person experiencing PTSD and encourage them to take care of the patient, not to discriminate him/her, to follow the doctor's instructions and help the person to reintegrate into the community.<br><br>If you wish to listen to this message again, press 1.
Closing	Please listen to the quiz.
Quiz/Test	Please answer one question related to today's lesson:<br><br>In case you've provided first aid to a person experiencing trauma and the situation does not improve, you should immediately take him/her to the nearest health center.<br><br>If it's Yes, press 1. if it's No, press 2.
Correct Answer	Thank you, you've responded well.
Incorrect Answer	Thank you for trying, the correct answer is "Yes"
No response	You have not responded to this question. Please listen to the question again and press a number that corresponds to your answer choice.

Conclusion	This is the end of today's lesson. After this lesson you will receive an SMS that summarizes what you have learned in today's session. If you wish to re-listen to this message or to share it with someone, you can beep this number [Phone number]. It is free of charge.<br><br>Thank you for listening to this message. Next time we will talk about depression.
Post call SMS (Key Messages)	Once you've provided first aid to a person experiencing trauma, you should urge him/her to consult a doctor and urge his/her family to take care of him/her. In case the condition gets more serious, immediately help him/her to get to the nearest health center.

Module 6 - Depression

**Segment**	**English translation of training message and in-module quiz question**
Advance SMS	Greetings. The Ministry of Health will call you tomorrow in the afternoon for training on depression. You will be called by [Phone number]. Thank you.
Introduction	Greetings! This is the important message from the Ministry of Health regarding training about mental health.<br><br>Listen carefully to the entire call and answer to the quiz.
Lesson Outcome	Did you know the symptoms of depression, the first aid to provide and how to support someone suffering from depression?
Narrative	Depression symptoms are severe sadness, insomnia, desperation, lack of happiness, giving up on life and suicide thoughts.<br><br>The first aid to provide to a depressive person is to be attentive to him/her, listening to him/her, comforting him/her, talking patiently and not judging him/her. Also helping his/her family to take good care of him/her and to support him by referring to the hospital.

	It is important to visit a family that has a person suffering from depression and recommend them to take care of the person, for the patient on treatment, help him/her take the right medication and to follow the doctor's instructions in order to avoid consequences of this condition such as committing suicide.<br><br>If you wish to listen to this message again, press 1.
Closing	Please listen to the quiz.
Quiz/Test	Please answer one question related to today's lesson:<br><br>Are isolation, lack of sleep and suicide thoughts some of the signs of a person suffering from depression?<br><br>If it's Yes, press 1. If it's No, press 2.
Correct Answer	Thank you, you've responded well.
Incorrect Answer	Thank you for trying, the correct answer is "Yes".
No response	You have not responded to this question. Please listen to the question again and press a number that corresponds to your answer choice.
Conclusion	This is the end of today's lesson. After this lesson you will receive an SMS that summarizes what you have learned in today's session. If you wish to re-listen to this message or to share it with someone, you can beep this number [Phone number]. It is free of charge.<br><br>Thank you for listening to this message. Next time we will talk about drug abuse.
Post call SMS (Key Messages)	A person suffering from depression is characterized by immense sorrow, lack of sleep, desperation, lack of happiness, giving up on life and suicide thoughts.

Module 7 - Drug Abuse

Segment	English translation of training message and in-module quiz question
Advance SMS	Greetings. The Ministry of Health will call you tomorrow afternoon for the training about drug abuse. You will be called by [Phone number]. Thank you.
Introduction	Greetings! This is the important message from the Ministry of Health regarding training about mental health.<br><br>Listen carefully to the entire call and answer to the quiz.
Lesson Outcome	Did you know what drug abuse is, signs that characterize the person using drugs and your role in fighting drug abuse and how you can support those addicted to drugs?
Narrative	A drug is a substance when taken can modify how someone thinks and takes decisions (examples: marijuana, illicit alcohol, sniffing glue, etc).<br><br>Some of the signs that show a person that has consumed drugs are excess of happiness for no reason, unusual boldness or indecency, unusual increased strength, etc.<br><br>Your role resides mostly in creating awareness around you on the negative consequences of drug abuse in order to fight the consumption and distribution of drugs (example: reporting and cooperation with other institutions). It is necessary to help a victim of drug abuse in taking him/her at the hospital with the support of his/her family.<br><br>If you wish to listen to this message again, press 1.
Closing	Please listen to the quiz.
Quiz/Test	Please answer one question related to today's lesson:<br><br>A drug is a substance if consumed by an individual modifies his/her ability to think and his/her comportment.<br><br>If it's Yes, press 1. If it's No, press 2.
Correct Answer	Thank you, you've responded well.

Incorrect Answer	Thank you for trying, the correct answer is “Yes”.
No response	You have not responded to this question. Please listen to the question again and press a number that corresponds to your answer choice.
Conclusion	This is the end of today’s lesson. After this lesson you will receive an SMS that summarizes what you have learned in today’s session. If you wish to re-listen to this message or to share it with someone, you can beep this number [Phone number]. It is free of charge.<br><br>Thank you for listening to this message. Next time we will talk about epilepsy.
Post call SMS (Key Messages)	Examples of drugs are marijuana, illicit alcohol, sniffing glue. Some of the signs that show a person that has consumed drugs are excess of happiness for no reason, unusual boldness or indecency, unusual increased strength, etc.

<u>Module 8 - Epilepsy</u>

**Segment**	**English translation of training message and in-module quiz question**
Advance SMS	Greetings. The Ministry of Health will call you tomorrow afternoon for the training about epilepsy. You will be called by [Phone number]. Thank you.
Introduction	Greetings! This is the important message from the Ministry of Health regarding training about mental health.<br><br>Listen carefully to the entire call and answer to the quiz.
Lesson Outcome	Did you know the symptoms of epilepsy, the basic first aid and how to educate the family members to take care of an epileptic patient?

Narrative	Epilepsy is a brain disorder. It’s not contagious. Some of its symptoms are loss of consciousness, falling down, stiffening of the body, biting of the tongue, foaming mouth, etc.<br><br>It is important to take care of a person that presents the above symptoms by aerating the place, taking him/her far for a fire or anything that could hurt him/her and also by staying next to him/her until he/she recovers consciousness.<br><br>You also have to immediately help him/her getting to the hospital. It’s also good to talk to him/her about taking the right medication as directed by the doctor. It is also necessary to engage with family members to avoid isolation of the sick person.<br><br>If you wish to listen to this message again, press 1.
Closing	Please listen to the quiz.
Quiz/Test	Please answer one question related to today’s lesson:<br><br>Epilepsy is a contagious disease.<br><br>If it's Yes, press 1. If it's No, press 2.
Correct Answer	Thank you, you've responded well.
Incorrect Answer	Thank you for trying, the correct answer is “No”.
No response	You have not responded to this question. Please listen to the question again and press a number that corresponds to your answer choice.
Conclusion	This is the end of today’s lesson. After this lesson you will receive an SMS that summarizes what you have learned in today’s session. If you wish to re-listen to this message or to share it with someone, you can beep this number [Phone number]. It is free of charge.

	Thank you for listening to this message. Next time we will ask you questions related to all that you have learned in this training.
Post call SMS (Key Messages)	For someone presenting symptoms of epilepsy, it’s important to take care of him by aerating his space, you also need to take him far from fire or anything that could hurt him. Please stay next to him until he regains consciousness.

Appendix 2: Baseline and Endline Quiz Questions

A. Baseline and endline questions:

1. What is a mental illness?
    a) Malaria
    b) Obesity
    c) Depression
    d) Not sure

2. What is an important cause of mental illness?
    a) Evil spirits
    b) Violence
    c) Malnutrition
    d) Not sure

3. What is the best way to support someone suffering from mental illness?
    a) Discipline them
    b) Pray for them
    c) Help the family to take them to the hospital
    d) Not sure

4. Can mental health diseases be passed on between people who spend a lot of time together?
    a) Yes
    b) No
    c) Not sure

5. Do you feel confident to refer people suffering from mental illness to the hospital?
    a) Yes
    b) No
    c) Not sure

6. Are you confident about doing community and family education of mental health issues?
    a) Yes
    b) No
    c) Not sure

B. Additional questions for the endline assessment only

7. Would you like to receive training in a similar way on other topics?
    a. Yes
    b. No
    c. Not sure

8. Would you recommend this training to others?
    a. Yes
    b. No
    c. Not sure
